# Genome Insights into Beneficial Microbial Strains Composing SIMBA Microbial Consortia Applied as Biofertilizers for Maize, Wheat and Tomato

**DOI:** 10.3390/microorganisms12122562

**Published:** 2024-12-12

**Authors:** Lisa Cangioli, Silvia Tabacchioni, Andrea Visca, Alessia Fiore, Giuseppe Aprea, Patrizia Ambrosino, Enrico Ercole, Soren Sørensen, Alessio Mengoni, Annamaria Bevivino

**Affiliations:** 1 Department of Biology, University of Florence, Sesto Fiorentino, 50121 Florence, FI, Italy; lisa.cangioli@unifi.it (L.C.); alessio.mengoni@unifi.it (A.M.); 2 Department for Sustainability, Italian National Agency for New Technologies, Energy and Sustainable Economic Development, ENEA Casaccia Research Center, 00123 Rome, RM, Italy; silvia.tabacchioni@enea.it (S.T.); andrea.visca@enea.it (A.V.); alessia.fiore@enea.it (A.F.); giuseppe.aprea@enea.it (G.A.); 3 AGRIGES srl, 82035 San Salvatore Telesino, BN, Italy; patrizia.ambrosino@agriges.com; 4 Centro Colture Sperimentali, CCS-AOSTA srl, 11020 Quart, AO, Italy; ercole.enrico@gmail.com; 5 Department of Biology, University of Copenhagen, DK-2200 Copenhagen, Denmark; sjs@bio.ku.dk

**Keywords:** biofertilizers, PGP bacteria, microbial consortia, whole-genome sequencing, risk assessment, traceability

## Abstract

For the safe use of microbiome-based solutions in agriculture, the genome sequencing of strains composing the inoculum is mandatory to avoid the spread of virulence and multidrug resistance genes carried by them through horizontal gene transfer to other bacteria in the environment. Moreover, the annotated genomes can enable the design of specific primers to trace the inoculum into the soil and provide insights into the molecular and genetic mechanisms of plant growth promotion and biocontrol activity. In the present work, the genome sequences of some members of beneficial microbial consortia that have previously been tested in greenhouse and field trials as promising biofertilizers for maize, tomato and wheat crops have been determined. Strains belong to well-known plant-growth-promoting bacterial genera such as *Bacillus*, *Burkholderia*, *Pseudomonas* and *Rahnella*. The genome size of strains ranged from 4.5 to 7.5 Mbp, carrying many genes spanning from 4402 to 6697, and a GC content of 0.04% to 3.3%. The annotation of the genomes revealed the presence of genes that are implicated in functions related to antagonism, pathogenesis and other secondary metabolites possibly involved in plant growth promotion and gene clusters for protection against oxidative damage, confirming the plant-growth-promoting (PGP) activity of selected strains. All the target genomes were found to possess at least 3000 different PGP traits, belonging to the categories of nitrogen acquisition, colonization for plant-derived substrate usage, quorum sensing response for biofilm formation and, to a lesser extent, bacterial fitness and root colonization. No genes putatively involved in pathogenesis were identified. Overall, our study suggests the safe application of selected strains as “plant probiotics” for sustainable agriculture.

## 1. Introduction

Plant-growth-promoting (PGP) bacteria are a taxonomically heterogeneous group of bacteria that can enhance plant growth and protect plants from disease and abiotic stress [1]. They are normally found within the plant-associated microbiota, as members of the rhizosphere or the phyllosphere (the habitats surrounding roots and leaves, respectively) or as endophytes of different plant organs. These bacteria can contribute to the host plant’s health and yield multiple abilities from the absorption of nutrients to the regulation of the host immune response [2]. They provide protection against pathogens, additional metabolic capabilities, essential nutrients and even phytohormone modulation [3].

In recent years, an ever-increasing interest in plant-associated bacteria has been manifested by researchers but also growers, consumers and policymakers. The effectiveness of PGP bacterial inoculants in contributing to a net increase in crop productivity coupled with a decrease in the use of agrochemicals has been demonstrated [4]. Interest in scouting plant-associated microbiota and culturing novel PGP bacteria is growing [5]. One of the main concerns with the utilization of inoculants composed of a single bacterial strain is that, while they may be effective under in vitro testing, they often fail in tests in field conditions, potentially due to competition with indigenous soil and plant microbiota, as well as variable environmental conditions [6,7]. Recently, innovative solutions have been proposed such as the production of microbial consortia composed of different species, which can have complementary and beneficial activities with respect to the plant and which can ensure enough persistence in the environment to produce reliable plant-growth-promoting effects [8,9,10]. Such microbial consortia, when mirroring the complexity and services of a native microbial community, are sometimes referred to as synthetic communities (SynComs) [11,12], and they comprise several members trying to mimic the core functions of a healthy native microbial community [13]. The choice of plant-growth-promoting microorganism (PGPM) is fundamental to developing efficient SynComs to be used as biofertilizers in agriculture, capable of boosting the growth of crop plants and exerting their biocontrol activity against phytopathogens. In many cases, SynComs have been tested under controlled conditions in pot experiments, providing the opportunity to model the complexity of plant–microbe interactions and of the soil and rhizosphere microbiome [14,15,16]. However, the discrepancy between laboratory results and performance on the field is often considerable, resulting in unsuccessful attempts at microbial application [17]. In order to mitigate the risk of failure of PGPM application in the field, a proposal for guidelines pertaining to the design and implementation of field trials was put forth [18]. The objective was to ensure reproducibility of the results, thereby preventing potential issues that could arise in the context of a future large-scale application.

The advent of next-generation sequencing (NGS) has facilitated the whole-genome sequencing (WGS) of several PGPRs, mainly isolated from crop species, such as *Pseudomonas* sp., *Bacillus* sp., *Paenibacillus polymyxa* and *Klebsiella* sp. [19,20,21,22,23,24]. Recently, the whole-genome study on endophytic *Bacillus velezensis* strains isolated from cultivated maize revealed the presence of numerous genes with significant roles in PGP and biocontrol activity against phytopathogens and unveiled multiple secondary metabolite biosynthetic gene clusters (BGCs) [25]. Also, a comprehensive WGS of *B. licheniformis* YB06, isolated from the rhizosphere soil of healthy *Codonopsis pilosula* plants, revealed a diverse array of genes linked to induced systemic resistance (ISR) and PGP traits [26]. Genome mining performed on a large panel of *B. ambifaria* strains revealed the presence of the biosynthetic gene cluster responsible for the production of cepacin, a metabolite involved in biocontrol activity against *Pythium* damping-off disease [27].

The identification of the potential pathways and genes of the soil/rhizosphere microorganisms related to plant growth promotion enables a better understanding of the molecular mechanisms of PGP and biocontrol activity, which is fundamental to developing more efficient biofertilizers. Furthermore, WGS offers the highest level of bacterial strain discrimination, facilitating the development of tools for traceability into the soil of SynComs and more targeted risk assessment to exclude any potential risk associated with scaling-up and commercial application [28,29]. Within the frame of the Horizon 2020 SIMBA project (Sustainable Innovation of Microbiome Applications in Food System), aimed at using beneficial microorganisms along the entire food production value chain, SynComs were developed as sustainable biofertilizers [9,30]. The efficacy and reproducibility of their application were evaluated in the greenhouse and under field conditions on various farms in Germany and Italy [31,32,33,34]. These experimental trials were conducted under both organic and conventional cultivation conditions, taking into account a variety of important crops like maize, wheat and tomatoes. In the present study, the whole-genome sequencing and annotation of some members of SIMBA SynComs, which had not previously been sequenced, and which had been successfully used in greenhouse and field studies, were performed, i.e., *Bacillus licheniformis* PS141, *Bacillus velezensis* BV84, *Burkholderia ambifaria* MCI 7, *Pseudomonas granadensis* A23/T3c, *Pseudomonas fluorescens* DR54 and *Rahnella aquatilis* BB23/T4d. In this study, we reported the genome sequencing of these strains, which allowed us to putatively identify the genetic basis of some plant-growth-promoting and biocontrol traits. Additionally, virulence and antibiotic resistance determinants were also inspected to evaluate the safety and risk to public health of such biofertilizers.

## 2. Materials and Methods

### 2.1. Bacterial Strains and Culture Conditions

A total of six strains were examined belonging to the species *Bacillus licheniformis*, *Bacillus velezensis*, *Burkholderia ambifaria*, *Pseudomonas fluorescens*, *Pseudomonas granadensis* and *Rahnella aqualitis*. The bacterial strains were isolated from soil, rhizosphere, or plant tissues. The isolation features and relevant information concerning their PGP activity have been previously reported [9] and are summarized in Table 1. The strains were recovered from glycerol stocks stored at −80 °C and streaked onto nutrient agar (NA) plates. After microbial growth at 28 °C for 24–48 h, 3–4 isolated colonies were transferred to 4 mL of nutrient broth (NB) medium and incubated overnight at 28 °C and 200 rpm.

### 2.2. Genome Sequences and Annotation

Genomic DNA from each microbial strain was extracted with sodium dodecyl sulfate proteinase K lysis buffer, followed by treatment with cetyltrimethylammonium bromide (CTAB) as described in *Current Protocols for Molecular Biology* [43]. The integrity of genomic DNA was evaluated through analysis on a 1% agarose gel and the quantity was determined through the use of a NanoDrop (NanoDrop Technologies, Wilmington, DE, USA) and a Qubit fluorometer (Invitrogen, Thermo Fisher Scientific, Waltham, MA, USA). Paired-end sequencing was conducted at IGA Technology Services (Udine, Italy) using the HiSeq 2000 Illumina platform with a 2 × 150 approach. For almost all species, drafts of the sequenced genomes of other strains were sequenced at 100× coverage; the strain *Bacillus velezensis* BV84, for which a sequenced genome was not available, was sequenced at a greater depth (200× coverage). The sequences were assembled with SPAdes [44]. Annotation and COG assignment were performed by Prokka 1.4.0 [45] from the Galaxy webserver Orione [46] (https://galaxyproject.org/use/orione/ (accessed on 12 September 2021)).

### 2.3. Genome Characterization and Mining

The taxonomic identification of the strains from the genome sequence was performed using the Type (Strain) Genome Server [47]. The dDDH values were computed using the Genome BLAST Distance Phylogeny approach. Formula *d4* from [48] was used since it is independent of genome length and is thus robust against the use of incomplete draft genomes. Secondary metabolite biosynthesis gene clusters in bacterial genomes have been identified by means of the AntiSMASH software tool [49] (https://antismash.secondarymetabolites.org/ (accessed on 1 October 2021)). The presence of type 3 secretion systems was analyzed by means of the integrated prediction pipeline for bacterial type 3 secreted effectors (T3Sepp) [50]. To perform pangenome analysis, the pipeline Roary [51] was used and core and dispensable/unique gene fractions against close relatives of each strain were identified.

The genomes were further annotated with RAST [52,53,54] and the predicted amino acid sequences of all strains were mapped against the plant growth promotion traits’ ontology with the PGPT-Pred tool [55,56,57], available on the web platform for plant-associated bacteria, PLaBAse (https://plabase.cs.uni-tuebingen.de/pb/plabase.php (accessed on 1 September 2024)).

### 2.4. Data Availability

Sequences are deposited in the NCBI database under BioProject ID PRJNA1018344 at the SRA Experiment SRX25324655.

## 3. Results

### 3.1. Genomic Sequencing Report

The genome size of the six sequenced bacterial strains of the SynCom ranged from 4.5 to 7.5 Mbp (Table 2). The number of genes spanned from 4402 to 6697, in agreement with the corresponding genome sizes.

For each genome, in silico DNA–DNA hybridization (DDH) and genome-based phylogeny were computed to allow a robust taxonomic classification of the strains [48]. The results of the taxonomic assignment based on dDDH values are reported in Table 3. Phylogenetic trees for each strain are shown in Supplementary Figure S1.

### 3.2. Prediction of Functions Related to Antagonism, Plant Growth Promotion and Virulence

The presence of functions related to antagonism, pathogenesis and other secondary metabolites that may be involved in plant growth promotion (i.e., siderophores) was inspected on the whole dataset of six strains by searching for secondary metabolite gene clusters and type 3 secretion systems. The results are reported in Table 4. As expected, many nonribosomal peptide synthetase (NRPS) gene clusters and genes involved in the biosynthesis of relevant secondary metabolites were found; in particular, for strains belonging to the *Bacillus*, *Pseudomonas* and *Burkholderia* genera, we identified genes coding for antibiotics, surfactants and siderophores. Most notably, NRPSs were found in all strains. *B. licheniformis* PS141 and *B. velezensis* BV84 were found harboring the largest number of NRPSs. Concerning PGP abilities, siderophore production gene clusters were found for *P. granadensis* A23/T3c, *P. fluorescens* DR54 and *R. aquatilis* BB23/T4d. Finally, several strains contained gene clusters for protection against oxidative damage (as for aryl polyene and carotenoid production) [58]. Finally, no strain was found to be harboring genes related to T3SSs (type 3 secretion systems).

Pangenome analysis of the six strains was performed by selecting four panels of strains with various degrees of phylogenetic relatedness (Supplementary Table S1 and Figure S1). This analysis, after the identification of unique genes for each strain (Supplementary Dataset S1), can facilitate the selection of gene candidates for primer design to allow the traceability of bioinoculants in the environment.

### 3.3. PGP Traits by Inferring Genome Sequences with PLaBAse Database

Mapping of the predicted amino acid sequences of all strains against the plant growth promotion traits’ ontology with the PGPT-Pred tool was performed. The total number of PGP traits identified in both the target and reference genomes is presented in Table 5. Interestingly, *Rahnella aquatilis* BB23/T4d exhibited the highest number of PGP traits among the target and reference genomes. Furthermore, *Bacillus licheniformis* PS141, *Bacillus velezensis* BV84, *Pseudomonas granadensis* A23/T3c and *Rahnella aquatilis* BB23/T4d were identified as having a greater number of PGP traits than those observed in the reference genomes.

**Figure 1:**
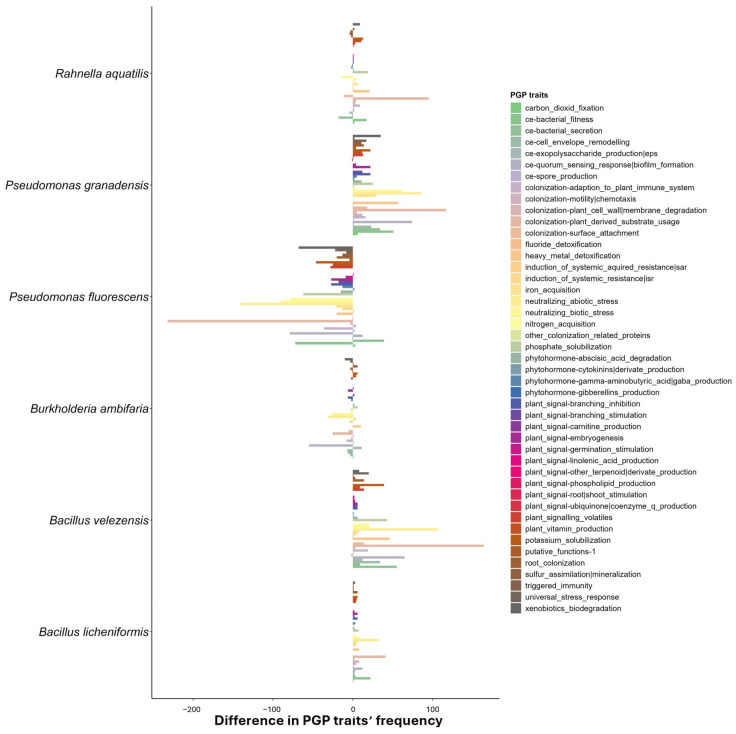
The difference in frequency occurrence of PGP traits (from PLaBAse) between the genomes presented in this work and the reference genome for each of them. A positive value represents an increase in the frequency occurrence of that PGP trait in the target genome, compared to the reference. A negative value represents a decrease in the frequency of occurrence of that PGP trait in the target genome, compared to the reference.

The difference between the reference and target genomes is reported in Figure 1. It is noteworthy that *P. granadensis* A23/T3c and *B. velezensis* BV84 exhibited the highest frequency of PGP traits, exceeding that of the reference strains. Despite *R. aquatilis* BB23/T4d reporting the highest number of PGP traits (Table 5), the difference in frequency between the target strain and the reference is minimal. Conversely, *P. fluorescens* DR54 was found to report lower-frequency values of PGP traits compared to the reference. Still, all the target genomes reported in this work were found to possess at least 3000 different PGP traits (Table 5).

Interestingly, the most prevalent PGP traits in the differential frequency plot (Figure 1) were identified as belonging to the categories of nitrogen acquisition, colonization for plant-derived substrate usage (e.g., indole-3 acetic acid degradation), quorum sensing response for biofilm formation and, to a lesser extent, bacterial fitness and root colonization.

A considerable number of PGP traits are grouped within these categories, as reported by Patz et al. [56].

## 4. Discussion

Whole-genome sequencing is an effective method to predict the safety of strains at the gene level, in terms of virulence and antibiotic resistance determinants, to identify genes that contribute to the beneficial activity of microbial strains and to select genetic markers that can enable tracing of the inoculum during field experiments. Under these premises, genome sequencing represents a relevant tool to allow the development of novel microbial inoculants.

All strains selected in this study were previously reported as PGPM strains (Table 1) and were selected as members of multifunctional microbial consortia (MC) according to their different modes of action, within the frame of the Horizon 2020 SIMBA project (Sustainable Innovation of Microbiome Applications in Food System) [10]. Their successful application in controlled environment conditions and open-field studies and the reproducibility of their efficacy on maize, wheat and tomato crops [10,31,32,33,34] allowed us to identify them as potential biofertilizers for sustainable agriculture. Despite the obvious agronomic advantages offered by biofertilization, it is mandatory to exclude any negative effects of their application on the behavior and genetic profile of soil microbial communities. It is well known that horizontal (or lateral) gene transfer (HGT) among bacterial species, one of the major mechanisms in bacterial evolution, could harbor virulence factors that could pose risks to human health [59]. In addition, whole-genome sequencing may be a helpful tool to track the establishment and/or persistence of specific microbes in the field and decipher the molecular and functional mechanisms of plant–microbe interaction [60].

Here, we investigated the WGS of six strains belonging to well-known plant-growth-promoting bacterial species including *Bacillus velezensis*, *Bacillus licheniformis*, *Burkholderia ambifaria*, *Pseudomonas granadensis*, *Pseudomonas fluorescens* and *Rahnella aquatilis* [9]. Genomic mining allowed for the exclusion of the presence of genes putatively involved in pathogenesis and identification of siderophore and NRPS gene clusters, which can explain the previous findings on strains’ abilities to possess multiple PGP traits and promote plant growth (see the references in Table 1). In addition, many gene clusters related to secondary metabolites involved in indirect mechanisms of plant growth have been detected in all sequenced genomes, suggesting a high potential of these strains as biocontrol agents. The presence of gene clusters involved in the protection against oxidative damage (as for aryl polyene and carotenoid production) [58] could contribute to strain survival in soil and in the bioinoculant formulation, as revealed in previous works performed in the greenhouse and in field trials [31,32,33,61].

Overall, genomic analysis revealed a broad spectrum of PGP genes in all strains, demonstrating significant functional diversity. The identification of PGP traits was conducted by inferring genome sequences using the PLaBAse database, and the highest number of PGP traits was detected in *Rahnella aquatilis* BB23/T4d, compared to the other strains. In a recent study [62], *Rahnella aquatilis* was found to improve plant growth and physiological parameters, indicating its potential for development as a biofertilizer to support a sustainable agricultural system [63,64]. The strain presented in this work, with its high number of PGP traits, proves essential for the consortium, as it was still detected one-month post-inoculation [61,65]. Concerning the other strains, they were found to harbor PGP traits, ranging from 3052 (*Bacillus licheniformis* PS141) to 3893 (*B. ambifaria* MCI 7). Genes associated with PGP traits such as “Plant Vitamin Production”, “Cell Envelope Remodeling”, “Universal Stress Response” and “Xenobiotics Biodegradation” are essential for rhizobacterial survival and plant nutrition, as they rely on plant exudates and contribute to plant growth by synthesizing metabolites and solubilizing nutrients, significantly impacting plant health and growth [66]. Also, the detection of genes related to quorum sensing and biofilm formation represents a favorable PGP trait for the establishment of strains on plant roots as a stage of the colonization process [65,67].

The data reported in this work could constitute the basis for developing a combined approach of phenotypic testing and genome sequencing of strains for the constitution of bioinoculant consortia. Prospectively, this approach can allow for the rational design of a SynCom formulation, which takes into account relevant genomic information, such as the putative biosynthetic pathways for relevant secondary metabolites and highlights the presence of a unique gene pool for each strain, which could be further used to design primers and develop molecular methods for biomonitoring (i.e., qPCR). In a recent study, based on in silico analysis and on the already published genomes of species composing the microbial consortium MC_C, developed within the frame of the Horizon 2020 SIMBA project, a real-time PCR (qtPCR) protocol was set up to follow the fate of PGPR strains under field conditions [61]. Results revealed that three out of the five bacterial species included in the MC_C consortium, i.e., *Burkholderia ambifaria*, *Bacillus amyloliquefaciens* and *Rahnella aquatilis*, were detected up to one month after inoculation, having been able to colonize and grow in the soil. In a previous work, from the whole-genome sequence of a *B. velezens* is strain NJAU-Z9, a strategy based on a real-time PCR method for directly monitoring of the target strain in the soil and the rhizosphere was developed [68]. Our annotated genomes will enable, therefore, the detection of specific DNA regions to be used for the design of unique and efficient quantitative primers for monitoring the target microorganisms composing beneficial microbial consortia, allowing the traceability of bioinoculants. Furthermore, the availability of the genome sequences of inoculated strains offers a strategy to evaluate the persistence of MC in the soil and/or rhizosphere, as well as the competitiveness of strains with indigenous microbiome members, by mapping metagenome sequence reads to the reference genomes [9].

## 5. Conclusions

In conclusion, the absence of pathogenicity traits, as well as the identification of genes that are likely responsible for the plant-growth-promoting and biocontrol features that were experimentally described, suggests that SIMBA microbial consortia represent good candidates for the scaling-up process and application of “plant probiotics” in sustainable agriculture. The exclusion of any pathogenic traits and the presence of a large plethora of PGP traits boost their use as microbial biofertilizers on a large scale. This study will enable us to proceed with the scaling-up and commercialization of SIMBA microbial consortia, which were highlighted as excellent innovations by the European Commission’s Innovation Radar team (https://simbaproject.eu/simba-partner-recognised-as-key-innovator/), (accessed on 12 April 2023). Our findings provide an essential contribution to build the necessary background for the safe application of microbiomes in food production.

## Figures and Tables

**Table 1:** Plant-growth-promoting microorganisms used in this study. The species names were verified according to the latest classification in the NCBI Taxonomy database.

PGPM	Strain	Origin	Country	Properties	References	Microbial Consortia/ Commercial Products
*Bacillus* *licheniformis*	PS141	Rhizosphere	Italy	Indole acetic acid (IAA) production	Unpublished results	SIMBA MC_A Bio-Semina PW (Agriges)
*Bacillus velezensis*	BV84	Endophyte Grape leaves	Italy	Biocontrol/PGP	Unpublished results	SIMBA MC_B MICOSAT F (CCS Aosta)
*Burkholderia* *ambifaria*	MCI 7	Maize rhizosphere	Italy	PGP	[35,36]	SIMBA MC_C
*Pseudomonas* *fluorescens*	DR54	Sugar beet rhizosphere	Denmark	Biocontrol	[37,38,39,40,41]	SIMBA MC_B, SIMBA MC_C
*Pseudomonas* *granadensis*	A23/T3c	Soil	Italy	PGP	[42]	SIMBA MC_A
*Rahnella aquatilis*	BB23/T4d	Soil	Italy	PGP	[42]	SIMBA MC_B, SIMBA MC_C

**Table 2:** Genome sequencing statistics and annotation.

Strains	Contigs	CDSs	Genes	Genome Size (bp)	rRNA *	tRNA *	tmRNA *	Repeat Region **
*Bacillus licheniformis* PS141	1242	4′318	4′402	4′504′417	6	77	1	/
*Bacillus velezensis* BV84	9′042	5′140	5′243	6′928′355	10	92	1	/
*Burkholderia ambifaria* MCI 7	274	6′618	6′697	7′509′419	4	74	1	/
*Pseudomonas granadensis* A23/T3c	1870	5′835	5′903	7′034′766	5	62	1	/
*Pseudomonas fluorescens* DR54	359	5′420	5′501	6′178′566	4	68	1	/
*Rahnella aquatilis* BB23/T4d	509	5′087	5′176	5′606′795	6	82	1	1

* Total number of ribosomal RNA (rRNA), transfer RNA (tRNA) and transfer-messenger RNA (tmRNA) genes. ** The sign / indicates absence of repeat regions.

**Table 3:** Taxonomic classification of strains.

Query Strain	Subject Strain	dDDH (*d4*, in %) *	C.I. (*d4*, in %)	G + C Content Difference (in %)
*Bacillus licheniformis* PS141	*Bacillus licheniformis* ATCC 14580	97.2	[96.1–98.0]	0.21
*Bacillus velezensis* BV84	*Bacillus velezensis* NRRL B-41580	83.8	[81.0–86.2]	3.3
*Burkholderia ambifaria* MCI 7	*Burkholderia ambifaria* AMMD	79.2	[76.3–81.9]	0.04
*Pseudomonas granadensis* A23/T3c	*Pseudomonas crudilactis* UCMA 17988T	47.1	[44.5–49.7]	0.45
*Pseudomonas fluorescens* DR54	*Pseudomonas* carnis B4-1T	77.7	[74.7–80.4]	0.23
*Rahnella aquatilis* BB23/T4d	*Rahnella aceris* SAP-19	95.7	[94.2–96.8]	0.13

* dDDH (formula *d4* along with confidence interval C.I.), and GC% difference with the best hit is shown.

**Table 4:** Presence of gene clusters linked to secondary metabolites and type 3 secretion systems.

Strains	NRPSs	Other Secondary Metabolite Predictions	T3SS
*Bacillus licheniformis* PS141	butirosin, fengycin, lichensyn, lassopeptide, surfactin, bacylisin, baciullibactin	terpene	0
*Bacillus velezensis* BV84	macrolactin, fengycin, piliplastin, difficidin, surfactin, bacylisin, baciullibactin, macrolactin	terpene	0
*Burkholderia ambifaria* MCI 7	T1PKS, NRPS	terpene, arylpolyene, phosphonate, pyrrolnitrin	0
*Pseudomonas granadensis* A23/T3c	NRPS, bacillomycin, pyoverdin, fragin	siderophore, terpene, arylpolyene	0
*Pseudomonas fluorescens* DR54	RiPP-like, pyoverdin, coelibactin, anikasin	siderophore, terpene, arylpolyene	0
*Rahnella aquatilis* BB23/T4d	lankacidin	betalactone,	0
		terpene,	
		arylpolyene	

NRPSs, nonribosomal peptide synthetases. T3SS, type 3 secretion system.

**Table 5:** Number of PGP traits found in the target and reference genomes.

Strain	Number of PGP Traits
*Bacillus licheniformis* PS141	3052
*Bacillus licheniformis* ATCC 14580 (reference genome)	2978
*Bacillus velezensis* BV84	3202
*Bacillus velezensis* FZB42 (reference genome)	2904
*Burkholderia ambifaria* MCI 7	3893
*Burkholderia ambifaria* AMMD (reference genome)	3904
*Pseudomonas fluorescens* DR54	3796
*Pseudomonas fluorescens* ATCC 13525 (reference genome)	4012
*Pseudomonas granadensis* A23/T3c	3877
*Pseudomonas granadensis* LMG 27940 (reference genome)	3650
*Rahnella aquatilis* BB23/T4d	4048
*Rahnella aquatilis* ATCC 33071 (reference genome)	4002

## Data Availability

Sequences were deposited in the NCBI database under BioProject ID PRJNA1018344 at the SRA Experiment SRX25324655.

## Supplementary Materials

The following supporting information can be downloaded at https://www.mdpi.com/article/10.3390/microorganisms12122562/s1, Table S1: Datasets of strains used for pangenome analysis; Figure S1: Genome-based phylogenies. Closest type strain genomes are included in each phylogeny as from Type (Strain) Genome Server pipeline [47]. Tree inferred with FastME 2.1.6.1 from GBDP distances calculated from genome sequences [69]. The branch lengths are scaled in terms of GBDP distance formula *d5*. The numbers above branches are GBDP pseudo-bootstrap support values > 60 % from 100 replications, with an average branch support of 91.4 %. The tree was rooted at the midpoint [70]; Supplementary dataset S1: A dataset containing a list of unique orthologs found in the genomes of the SynCom and pangenome statistics and results obtained. Refs. [69,70] can be found in Supplementary Materials.
