# Supplementary material for: Genome Insights into Beneficial Microbial Strains Composing SIMBA Microbial Consortia Applied as Biofertilizers for Maize, Wheat and Tomato
Source: Microorganisms. 2024 Dec 12;12(12):2562. doi: 10.3390/microorganisms12122562 (PMC11677507; doi:10.3390/microorganisms12122562)
Supplement: Supplementary file 1 [file microorganisms-12-02562-s001.zip › Cangioli et al. Microorganisms_Supplementary Materials_revised.pdf]

Supplementary Materials

Genome Insights into Beneficial Microbial Strains Composing SIMBA Microbial Consortia Applied as Biofertilizers for Maize, Wheat and Tomato

Lisa Cangioli [1], Silvia Tabacchioni [2], Andrea Visca [2], Alessia Fiore [2], Giuseppe Aprea [2], Patrizia Ambrosino [3], Enrico Ercole [4], Soren Sørensen [5], Alessio Mengoni [1] and Annamaria Bevivino [2,*]

[1] Department of Biology, University of Florence, Sesto Fiorentino, 50121 Florence, FI, Italy; lisa.cangioli@unifi.it (L.C.); alessio.mengoni@unifi.it (A.M.)
[2] Department for Sustainability, Italian National Agency for New Technologies, Energy and Sustainable Economic Development, ENEA Casaccia Research Center, 00123 Rome, RM, Italy; silvia.tabacchioni@enea.it (S.T.); andrea.visca@enea.it (A.V.); alessia.fiore@enea.it (A.F.); giuseppe.aprea@enea.it (G.A.)
[3] AGRIGES srl, 82035 San Salvatore Telesino, BN, Italy; patrizia.ambrosino@agriges.com
[4] Centro Colture Sperimentali, CCS-AOSTA srl, 11020 Quart, AO, Italy; ercole.enrico@gmail.com
[5] Department of Biology, University of Copenhagen, DK-2200 Copenhagen, Denmark; sjs@bio.ku.dk
* Correspondence: annamaria.bevivino@enea.it; Tel.: +39-335-7664187 or +39-06-30483868

Supplementary Table S1. Datasets of strains used for pangenome analysis.

Supplementary Figure S1. Genome-based phylogenies. Closest type strain genomes are included in each phylogeny as from Type (Strain) Genome Server pipeline [47]. Tree inferred with FastME 2.1.6.1 from GBDP distances calculated from genome sequences [75]. The branch lengths are scaled in terms of GBDP distance formula *d5*. The numbers above branches are GBDP pseudo-bootstrap support values > 60 % from 100 replications, with an average branch support of 91.4 %. The tree was rooted at the midpoint [76].

Supplementary Dataset S1. A dataset containing the list of unique orthologs found in the genomes of the SynCom and pangenome statistics and results obtained.

Supplementary Table S1. Datasets of strains used for pangenome analysis.

**Dataset 1**	
Azotobacter chrococcum	*Burkholderia ambifaria*
Azotobacter vinelandii	*Paraburkholderia tropica*
Bacillus amyloliquefaciens	*Pseudomonas granadensis*
***Bacillus velezensis* BV84**	*Pseudomonas fluorescens*
Bacillus licheniformis	*Rahnella acquatilis*
**Dataset 2**	
Azotobacter chrococcum	*Pseudomonas putida*
Azotobacter vinelandii	*Rahnella contaminans*
Burkholderia ambifaria	*Serratia marcescens*
Paraburkholderia tropica	*Azotobacter armeniacus*
Pseudomonas granadensis	*Pseudomonas brassicacearum*
Pseudomonas fluorescens	*Enterobacter chloacae*
Rahnella aquatilis	*Burkholderia vietnamensis*
Ralstonia solanacearum	*Burkholderia cenocepacia* AU 1054
**Dataset 3**	
Bacillus subtilis	*Bacillus anthracis*
Baciullus amyloliquefaciens	*Bacillus albus*
***Bacillus velezensis* BV84**	*Bacillus cereus* ATCC 4342
Bacillus licheniformis	*Bacillus turingensis*

Supplementary Figure S1. Genome-based phylogenies. Closest type strain genomes are included in each phylogeny as from Type (Strain) Genome Server pipeline [47]. Tree inferred with FastME 2.1.6.1 from GBDP distances calculated from genome sequences [75]. The branch lengths are scaled in terms of GBDP distance formula *d5*. The numbers above branches are GBDP pseudo-bootstrap support values > 60 % from 100 replications, with an average branch support of 91.4 %. The tree was rooted at the midpoint [76].

***Bacillus licheniformis* PS141**

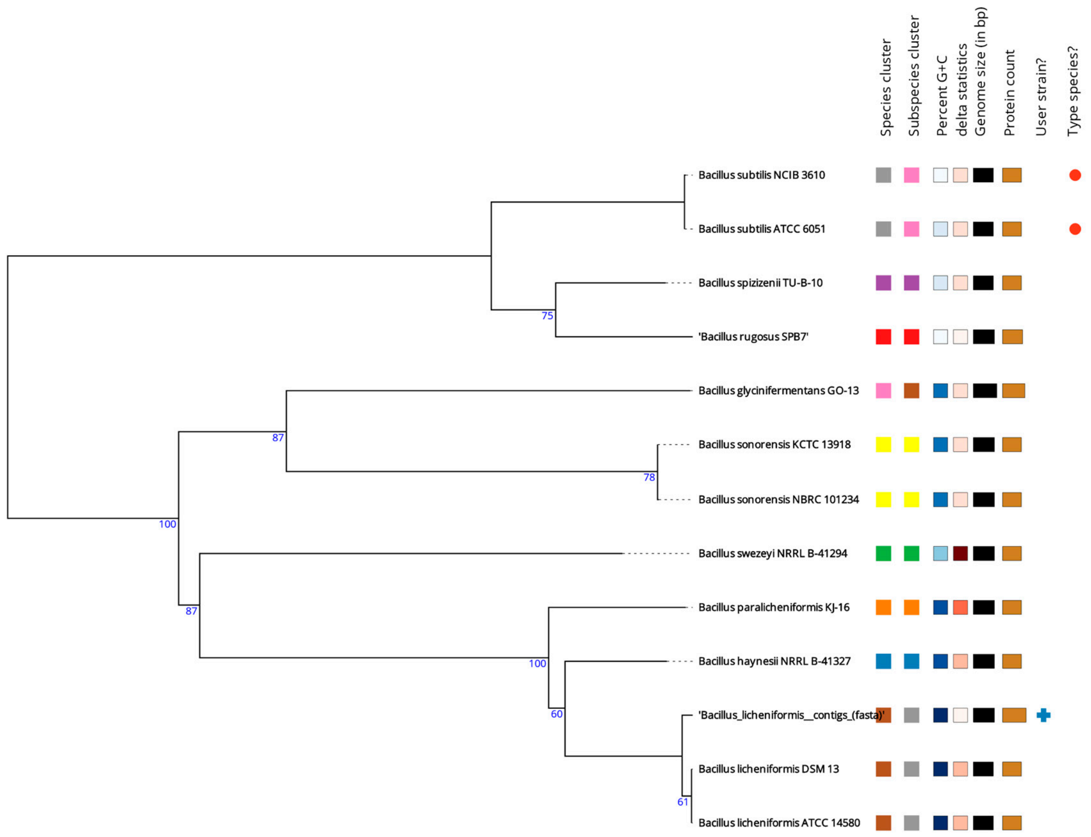

Bacillus velezensis BV84

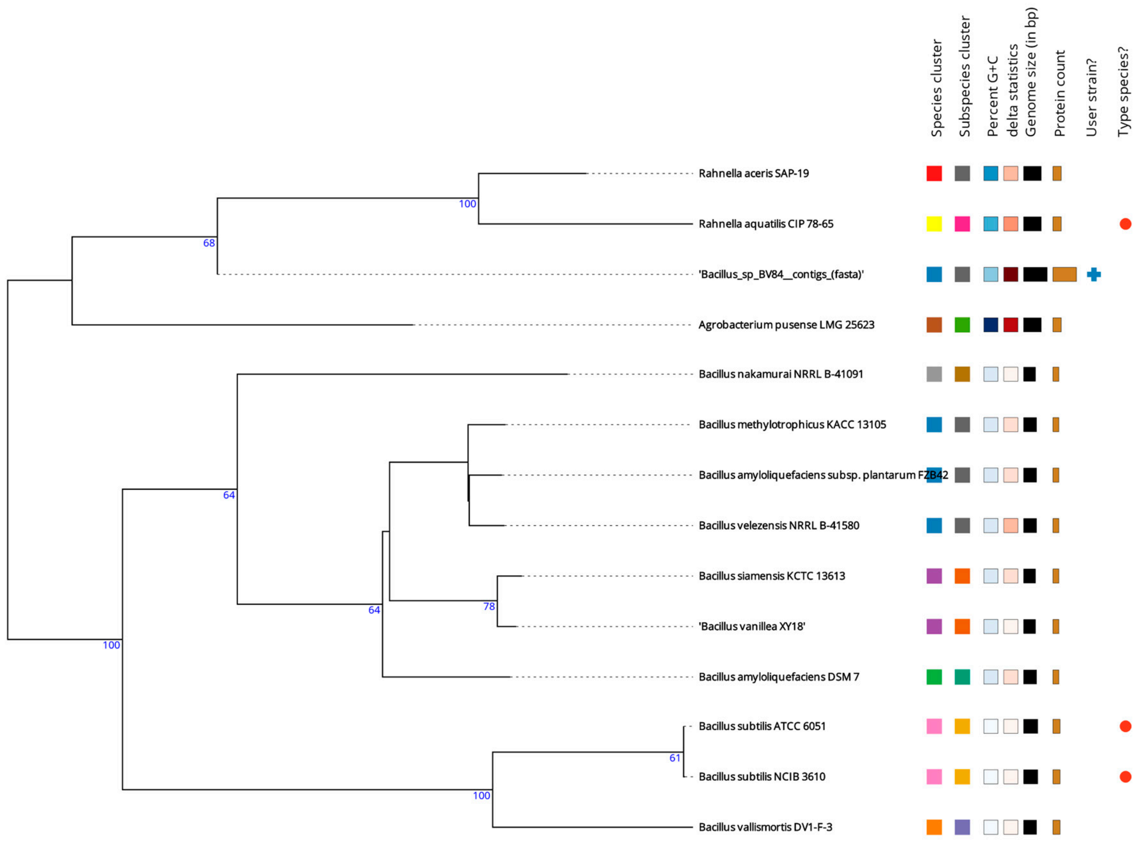

Burkholderia ambifaria MCI 7

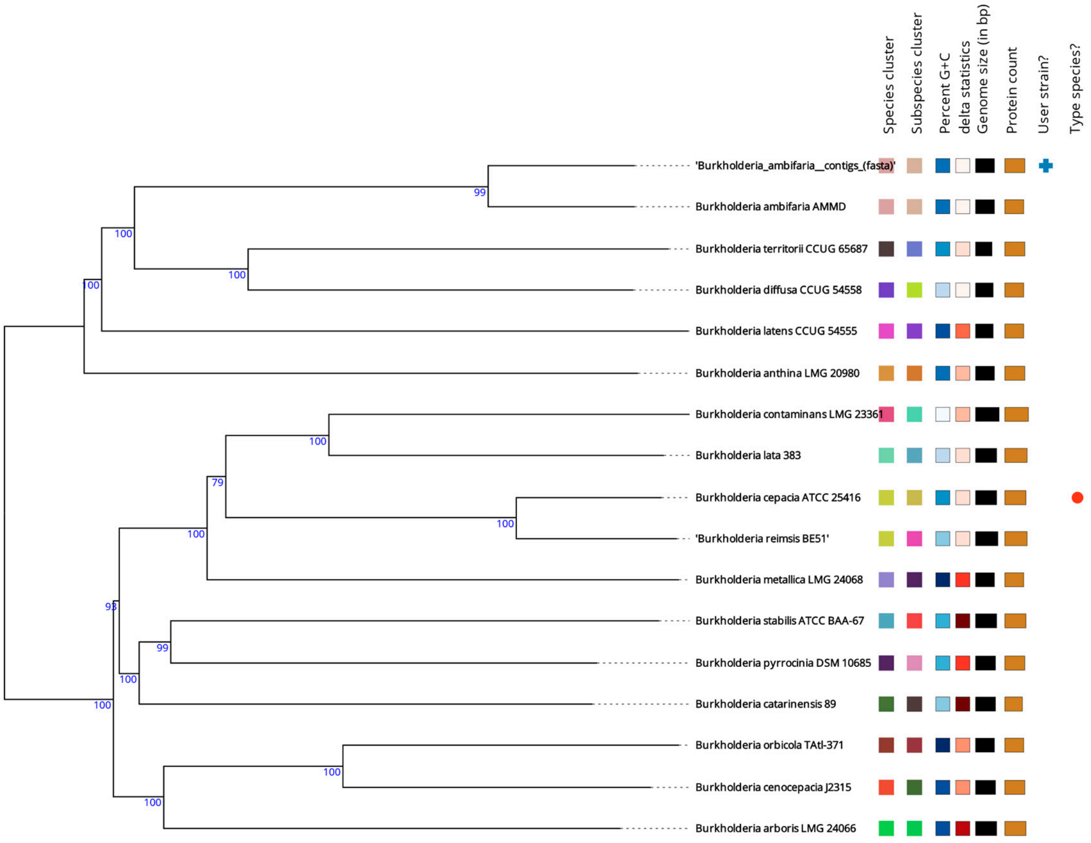

Pseudomonas fluorescens DR54

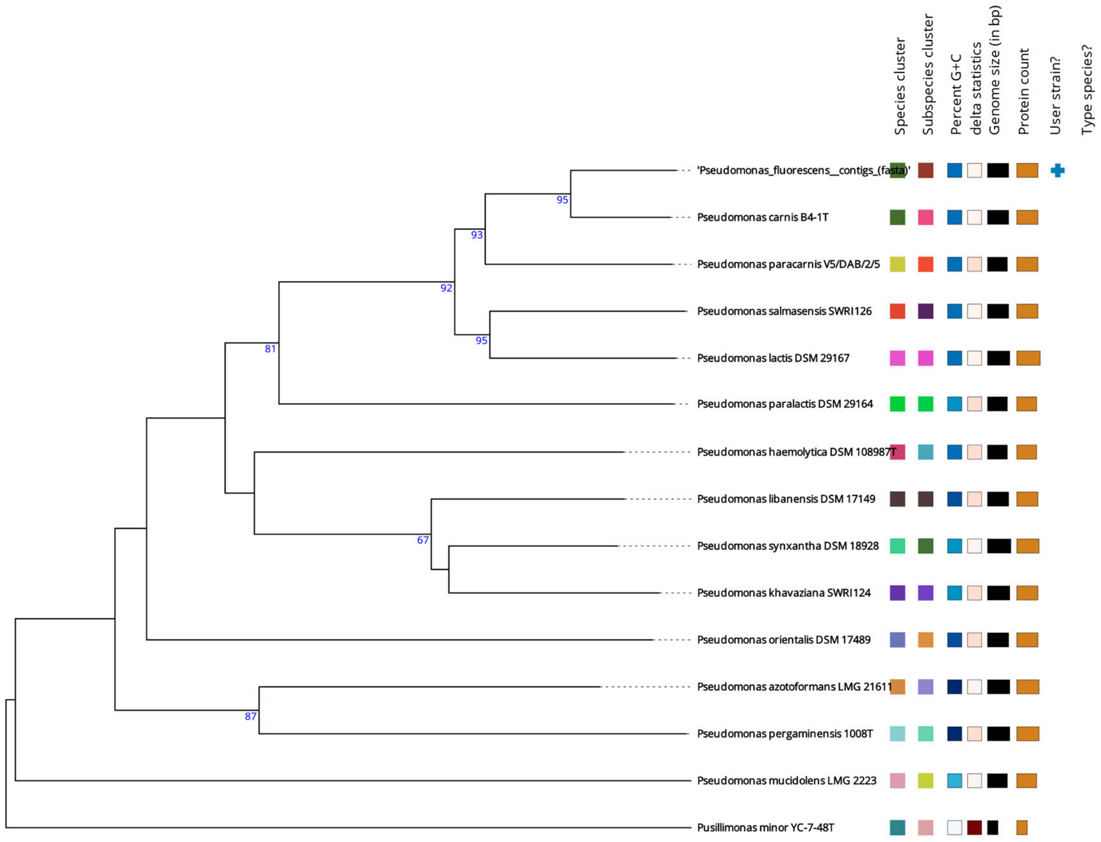

Pseudomonas granadensis A23/T3c

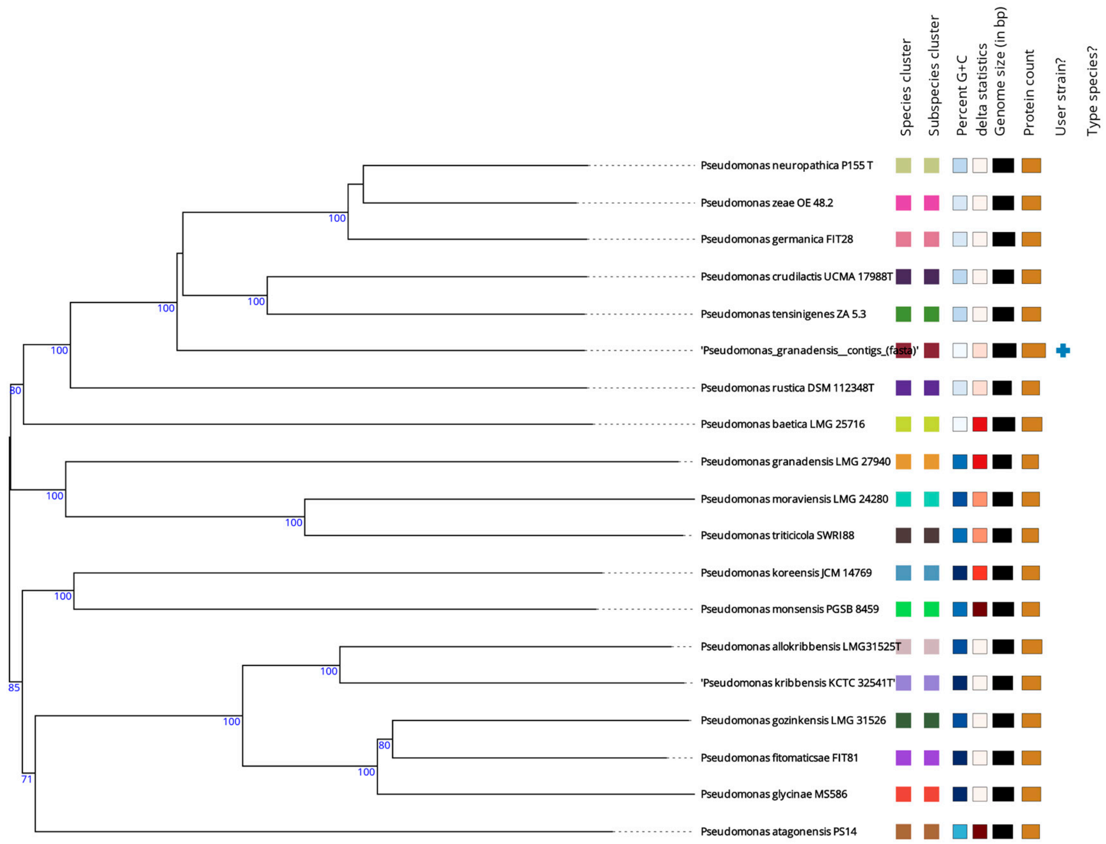

Rahnella aquatilis BB23/T4d

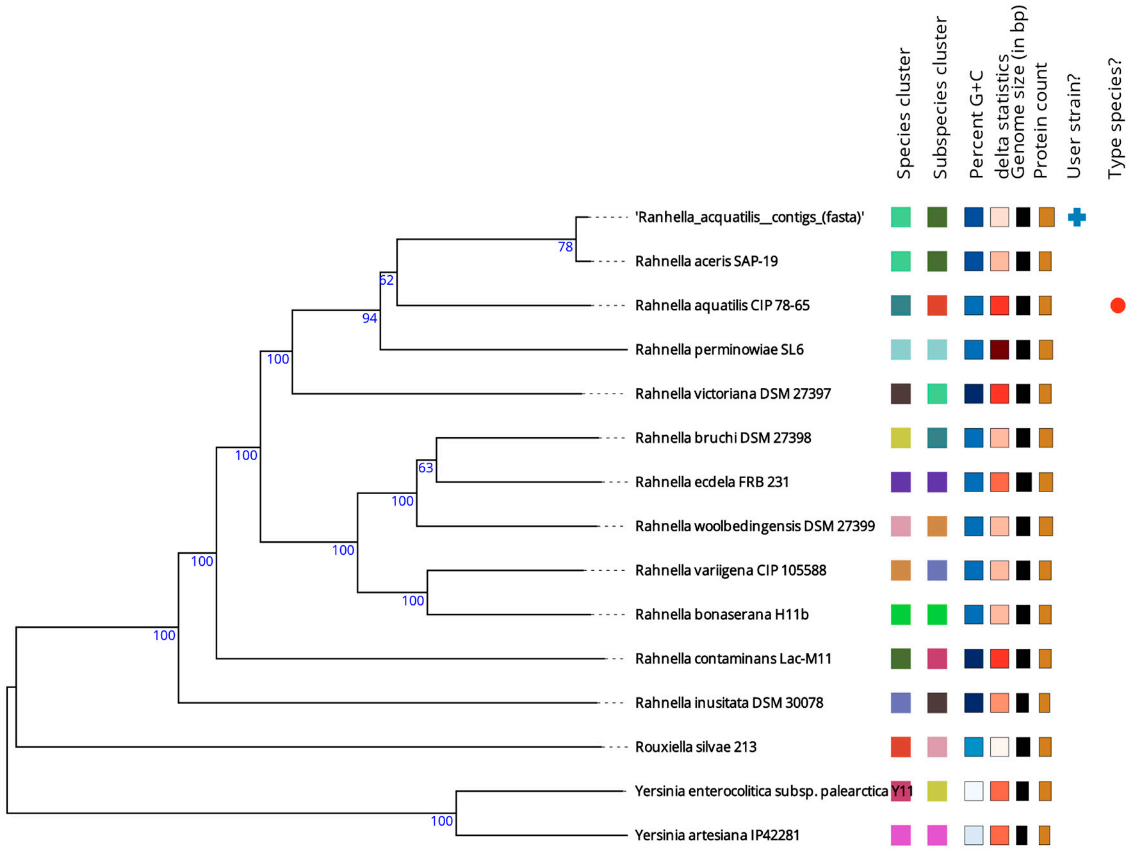

References

69 Lefort, V.; Desper, R.; Gascuel, O. FastME 2.0: A comprehensive, accurate, and fast distance-based phylogeny inference program. *Mol Biol. Evol.* **2015**, *32*, 2798–2800. https://doi.org/10.1093/molbev/msv150

70 Farris, J.S. Estimating Phylogenetic Trees from Distance Matrices. *Am. Nat.* **1972**, *106*, 645–668. https://doi.org/10.1086/282802
